# The Impact of Systemic Health Parameters on Intraocular Pressure in the Western Region of Saudi Arabia

**DOI:** 10.7759/cureus.25217

**Published:** 2022-05-22

**Authors:** Nawaf Almarzouki, Sumayya A Bafail, Daniyah H Danish, Sultan R Algethami, Noorah Shikdar, Saif Ashram, Tala Roblah

**Affiliations:** 1 Ophthalmology, King Abdulaziz University Faculty of Medicine, Jeddah, SAU; 2 Medicine, King Abdulaziz University Faculty of Medicine, Jeddah, SAU; 3 Internal Medicine, King Abdulaziz University Faculty of Medicine, Jeddah, SAU

**Keywords:** body mass index, glaucoma, hypertension, diabetes, intraocular pressure

## Abstract

Background

A normal intraocular pressure (IOP) is essential for maintaining the normal structure and function of the eyes. Furthermore, an elevated IOP is a known risk factor for glaucoma. As the results from studies addressing the relationship between IOP and systemic health parameters are conflicting, researchers have not reached a consensus. This study aimed to evaluate the relationship between IOP and health parameters among non-glaucomatous participants in the western region of Saudi Arabia.

Method

We retrospectively reviewed the medical records of 255 participants aged 20 years and above who had not received any medical treatment for ocular hypertension or glaucoma at King Abdulaziz University Hospital (KAUH), Jeddah, Saudi Arabia, from January 2019 to April 2021. The patients were categorized into age groups, divided by decades (ranging from 20-29 years to 80+ years); the most prevalent age group was 60-69 years. The data were entered using Microsoft Excel 2016 (Microsoft Corporation, Redmond, Washington), and Statistical Package for the Social Sciences (SPSS) software was used for univariate analysis. The relationship between continuous variables was analyzed by the Pearson correlation coefficient. The differences between continuous and categorical variables were assessed by the t-test and one-way analysis of variance (ANOVA) test, respectively.

Results

The mean (standard deviation) IOP in the right and the left eyes was 15.7 mmHg (4.0) and 15.6 mmHg (3.9), respectively. There were no significant associations between IOP and lipid profile parameters (p > 0.05). There was a statistically significant difference in the right IOP, in relation to the age groups (p = 0.006). Moreover, the mean IOP in the left eye was significantly higher among patients with diabetes than in the patients without diabetes (p = 0.007) as well as in patients with hypertension than in the patients without hypertension (p = 0.023).

Conclusion

The effect of total cholesterol, triglyceride (TG), low-density lipoprotein (LDL), high-density lipoprotein (HDL), BMI, and sex on IOP could not be established in our study. However, over the past years, people's diets have incorporated higher cholesterol and fat content, leading to higher BMI levels. Therefore, further studies of the association between BMI and IOP are critical to determine if BMI is certainly a significant risk factor for increased IOP and glaucoma.

## Introduction

Glaucoma is a type of progressive optic neuropathy, which is characterized by the degeneration of retinal ganglion cells and resulting changes in the optic nerve head [1]. Worldwide, glaucoma is the leading cause of permanent blindness [2]. Furthermore, it was estimated that 57.5 million developed blindness due to open-angle glaucoma [3]. The only modifiable risk factor for glaucoma is intraocular pressure (IOP) [2]. A normal IOP is maintained by balancing the aqueous humor production by the ciliary body with aqueous humor outflow via Schlemm's canal and uveoscleral tissues that drain into ophthalmic veins [4]. An IOP greater than 21 mmHg has been considered a criterion for the existence of glaucoma [5]. The IOP is influenced by various factors such as systemic health parameters and racial differences [6-10]. Knowledge of the factors affecting IOP would aid physicians in preventing the development of glaucoma [11].

Several epidemiological studies in Eastern populations have found a link between systemic health parameters and IOP [10,12,13]. For example, a positive correlation between IOP and age was found in Pakistani and Western populations [12], while a negative correlation between IOP and age was seen in Japanese, Taiwanese, and Korean populations [10,12-14]. An individual’s sex also has an effect on the IOP; a study found that the positive correlation between IOP and age was more significant in females than in males [8,10].

In addition to age and sex, previous studies have also investigated the presence of associations between IOP and the body mass index (BMI), hypertension, and abnormal lipid profile parameters [15-18]. The Beaver Dam Eye Study showed that reduced IOP is linked to lower systemic blood pressure [19]. Similarly, the Baltimore Longitudinal Study of Aging reported a positive correlation between systolic blood pressure (SBP) and IOP [20]. Furthermore, a study conducted by Lee et al. demonstrated a positive relation between IOP and BMI in Korean males, but no relation between BMI and IOP in Korean females [21].

On the other hand, Stewart et al. have found that increased levels of total cholesterol and high-density lipoprotein (HDL) are not associated with an increase in IOP [22]. These studies provide controversial and conflicting evidence on the association between hypertension, age, sex, and hyperlipidemia with IOP [15]. To the best of our knowledge, studies that address the effect of systemic health parameters on IOP have not been conducted in the western region of Saudi Arabia. In this study, we aim to assess the relationship between IOP and age, sex, BMI, SPB, diastolic blood pressure (DBP), diabetes, hypertension, total cholesterol, low-density lipoprotein (LDL), HDL, and triglyceride (TG) levels.

## Materials and methods

Materials and methods

This was a tertiary center retrospective record review study conducted in the ophthalmology department of King Abdulaziz University Hospital (KAUH), Jeddah, from June 2021 to July 2021.

Outcomes

The primary outcomes were the IOP levels in the right and left eyes compared to the levels of total cholesterol, HDL, LDL, and triglyceride. The secondary outcomes were the IOP levels in the right and left eyes compared to age, sex, BMI, SBP, DBP, hypertension, and diabetes.

Sample size and data collection methods

A total of 1401 medical records of people who visited the ophthalmology clinic from January 1, 2019, to April 30, 2021, were reviewed retrospectively, and 255 subjects were included in this study. Inclusion criteria were adult patients above 20 years of age with no previous chronic ocular disease especially (glaucoma). Exclusion criteria were subjects who had used topical or systemic steroids for a period of more than two months, subjects diagnosed with glaucoma or ocular hypertension, and subjects who had undergone any intraocular surgery. The following data were extracted from the medical record of each patient: age, sex, total cholesterol, HDL, LDL, and TG levels, SBP, DBP, diabetes, hypertension, and BMI. The BMI was calculated by the following equation: $BMI = \frac{Weight\ (kg)}{Height^{2}\ (m)}$. Blood pressure was measured by using a sphygmomanometer and the average of both arms was calculated.

Data entry and data analysis

The Microsoft Excel 2016 software (Microsoft Corporation, Redmond, Washington) was used for entering the data. Statistical analysis was performed using the Statistical Package for the Social Sciences (SPSS) software version 26.0 (IBM Corp., Armonk, NY). Continuous variables were represented by measures of central tendency, while categorical variables were represented by percentages and numbers. The relationship between continuous variables was analyzed by the Pearson correlation coefficient. The differences between continuous and categorical variables were assessed by the t-test and one-way analysis of variance (ANOVA) test, respectively. A p-value < 0.05 was considered significant.

Ethical approval

The study has been approved by the KAUH Institutional Review Board (Reference No. 125-21) and was conducted in accordance with the principles of the Helsinki Declaration.

## Results

A total of 255 participants were included. Subjects' descriptive and demographic data are shown in Tables 1, 2.

**Table 1:** Subjects' descriptive data IOP: Intraocular pressure; Rt IOP: Right eye intraocular pressure; Lt IOP: Left eye intraocular pressure; SD: Standard deviation; HDL: High-density lipoprotein; LDL: Low-density lipoprotein; BMI: Body mass index.

Parameters	Mean [SD]	Range
Age (years)	57.6 [14.5]	21-104
Systolic blood pressure (mmHg)	136.6 [20.6]	94-228
Diastolic blood pressure (mmHg)	73.8 [12.7]	46-123
BMI (kg/m^2^)	30.3 [6.6]	15.9-58.6
Rt IOP (mmHg)	15.7 [4.0]	7-31
Lt IOP (mmHg)	15.6 [3.9]	7-36
Total cholesterol (mg/dl)	81.3 [28.2]	36.7-268.5
HDL (mg/dl)	22.6 [6.4]	10-4.2
LDL (mg/dl)	54.7 [28.1]	18.7-217.6
Triglyceride (mg/dl)	27.4 [16.1]	5.9-118.9

**Table 2:** Subjects' demographic data (n = 255)

Descriptive	Number	Percentage (%)
Sex		
Female	142	55.7
Male	113	44.3
Age group (years)		
20-29	11	4.3
30-39	20	7.8
40-49	38	14.9
50-59	58	22.7
60-69	75	29.4
70-79	43	16.9
≥80	10	3.9
Nationality		
Saudi	190	74.8
Non-Saudi	65	25.2
Hypertension		
Hypertensive	96	37.8
Non-hypertensive	158	62.2
Diabetes		
Diabetic	157	61.8
Non-diabetic	97	38.2

Table 3 shows the Pearson correlation coefficients for IOP compared with systemic health parameters. There were no statistically significant correlations between IOP and total cholesterol (p = 0.344 for right eye intraocular pressure [Rt IOP] and p = 0.943 for left eye intraocular pressure [Lt IOP]), IOP and HDL (p = 0.579 for Rt IOP and p = 0.348 for Lt IOP), IOP and LDL (p = 0.937 for Rt IOP and p = 0.863 for Lt IOP) as well as IOP and TG (p = 0.529 for Rt IOP and p = 0.223 for Lt IOP).

**Table 3:** Univariate analysis for correlations between IOP and systemic health parameters R: Correlation coefficient; p: Statistical significance; IOP: Intraocular pressure; Rt IOP: Right eye intraocular pressure; Lt IOP: Left eye intraocular pressure; SBP: Systolic blood pressure; DBP: Diastolic blood pressure; BMI: Body mass index; HDL: High-density lipoprotein; LDL: Low-density lipoprotein. The Pearson correlation test was used for this analysis.

Variables	RT IOP		LT IOP	
	R-value	p-value	R-value	p-value
Age	+0.001	0.990	0.000	0.997
SBP (mmHg)	+0.107	0.112	+0.087	0.195
DBP (mmHg)	+0.026	0.699	+0.027	0.685
BMI (kg/)	+0.055	0.412	+0.022	0.748
Total cholesterol (mg/dL)	+0.075	0.344	+0.006	0.943
HDL (mg/dL)	-0.057	0.579	-0.096	0.348
LDL (mg/dL)	-0.007	0.937	-0.015	0.863
Triglyceride (mg/dL)	+0.050	0.529	+0.097	0.223

Table 4 shows the mean values of IOP specific for sex, diabetes, and hypertension. There were no significant associations in both Rt IOP and Lt IOP between males and females (p = 0.244 and p = 0.559, respectively). Similarly, no significant difference in Rt IOP was found between participants with and without diabetes (p = 0.093) or between participants with and without hypertension (p = 0.341). However, the mean Lt IOP for patients with diabetes (16.25 mmHg) was significantly higher than that for patients without diabetes (14.8 mmHg) (p = 0.007). The mean Lt IOP was also significantly higher in participants with hypertension (16.44 mmHg) than in those without hypertension (14.80 mmHg) (p = 0.023).

**Table 4:** Univariate analysis for the associations between IOP and sex, diabetes, and hypertension Rt: Right; Lt: Left; IOP: Intraocular pressure. The independent t-test was used for this analysis. *The right and left IOPs are represented as the mean [standard deviation].

Variables		Rt IOP	p-value	Lt IOP*	p-value
		Mean [SD]		Mean [SD]	
Sex	Female	16.03 [3.98]	0.244	15.53 [3.18]	0.559
	Male	15.40 [4.00]		15.86 [4.75]	
Diabetes	Diabetic	16.11 [4.09]	0.093	16.25 [4.29]	*0.007*
	Non-diabetic	15.20 [3.80]		4.80 [3.18]	
Hypertension	Hypertensive	16.08 [3.88]	0.341	16.44 [4.15]	*0.023*
	Non-hypertensive	15.20 [3.80]		14.80 [3.18]	

Table 5 shows the association between IOP and the age group as well as IOP and the BMI group based on the ANOVA test. The results showed that the association between Rt IOP and the age group is statistically significant [F(6,220) = 3.146, p = 0.006]. On the other hand, the association between Lt IOP and age was not statistically significant [F(6,219) = 1.898, p = 0.082]. Likewise, there was no statistically significant association between IOP and the BMI group ([F(3,23) = 2.211, p = 0.088 for Rt IOP] and [F(3,222) = 1.207, p = 0.308 for Lt IOP]).

**Table 5:** Univariate analysis for the associations between IOP, age groups, and BMI IOP: Intraocular pressure; BMI: Body mass index; p: Statistical significance. The analysis of variance (ANOVA) test was used for this analysis.

Variables		Right IOP (mmHg)	P-value	Left IOP (mmHg)	P-value
		Mean [SD]		Mean [SD]	
Age (years)	20-29	16.22 [4.94]	*0.006*	15.11 [3.78]	0.082
	30-39	13.80 [3.60]		14.75 [3.62]	
	40-49	14.96 [3.27]		15.09 [3.43]	
	50-59	16.99 [4.49]		16.57 [4.40]	
	60-69	16.48 [3.94]		16.40 [4.16]	
	70-79	14.29 [3.34]		14.47 [3.32]	
	≥80	14.75 [3.10]		14.37 [2.92]	
BMI	Underweight	10 [2]	0.088	11.66 [2.88]	0.308
	Healthy weight	15.96 [3.52]		16.10 [4.65]	
	Overweight	15.96 [3.17]		15.59 [3.12]	
	Obese	15.71 [4.52]		15.68 [4.10]	

## Discussion

The aim of this study is to determine the effect of systemic health parameters on IOP in the western region of Saudi Arabia and elaborate on any relation to previous studies.

Age and IOP 

In this study, we found a statistically significant association between Rt IOP and the age group, but there was no significant association between the Lt IOP and the age group. Previous studies have shown significant positive relationships between IOP and age in populations from Western regions, Pakistan, Italy, and the United States [23-29]. Conversely, some studies have shown a significant negative relationship between IOP and age in Japanese, Taiwanese, and Korean populations [10,13,30]. Other studies have found no significant association between IOP and age [31]. These variations could be due to racial differences, lifestyles, environmental factors, instruments used to measure IOP, and the criteria for exclusion of participants [12].

Sex and IOP

Our study found no significant association between either Rt or Lt IOP and sex. Some studies have shown no significant association between IOP and sex in American, Iranian, Korean, and Japanese populations [16,28,32,33]. In contrast, some studies have reported a significant association between IOP and sex. Some of these studies showed higher IOP values in men [26,27], while others showed higher IOP values in women [13,34]. Gender-related differences in IOP could be attributed to hormonal influences, SBP, total cholesterol and TG levels, and BMI [35].

BMI and IOP

In this study, BMI and IOP measurements were not significantly correlated. Similar to our findings, the Blue Mountain Eye Study and a study conducted by Han et al. found no significant relationship between IOP and BMI [12,36]. On the contrary, most other studies have found a significant positive association between IOP and BMI. For instance, a study by Hoehn et al. found that in women, the IOP increased with an increase in BMI [37]. Furthermore, a study by Han et al. found a positive association between IOP and BMI in both sexes [12]. This positive correlation could be due to the presence of excess fat tissue, which raises the episcleral venous pressure leading to a decrease in the outflow [29]. These apparently conflicting results regarding whether an association exists between IOP and BMI could be attributed to differences in methodologies, ethnicities, and instruments used to measure IOP. For example, Goldmann's applanation tonometer overestimates IOP in obese women [37]. The relationship between IOP and BMI should be studied further, since the BMI is an objective measure of obesity, along with the current obesity epidemic for preventing the development of glaucoma [38].

Lipid profile and IOP

Even though there was no statistically significant link in our study between the lipid profile and IOP in either eye, the IOP was lower in subjects with higher HDL levels. However, the other lipid profiles were positively correlated with IOP. A previous study from Japan has shown a significant association between Rt IOP and both total cholesterol and TG as well as a Lt IOP association only with TG. Moreover, it showed a positive correlation with all lipid profiles, except HDL, which was negatively correlated [35]. Another study from Korea showed a significant association between the IOP from both eyes and cholesterol and TG levels but no significant association with HDL. The same study showed that in men, HDL was positively correlated with IOP, while in women, it was inversely or negatively correlated with IOP [12]. The results from a study from Taiwan demonstrate a significant association between TG and IOP of both eyes in men only, while the rest of the parameters were non-significant. All other lipid profile parameters had a positive correlation with IOP [31].

Our study did not uncover a link between IOP and lipid profile that was statistically significant. This could be due to demographic differences, and most patients with hypercholesterolemia and hypertriglyceridemia will seek additional treatment in general physician clinics or dedicated metabolic clinics. As a result, identifying a substantial link between IOP and hypercholesterolemia or hypertriglyceridemia in our study sample would be difficult.

IOP diabetes and hypertension 

For the associations between IOP, diabetes, and hypertension, patients with diabetes had significantly higher IOP values compared to patients without diabetes. Moreover, patients with hypertension had significantly higher IOP compared to patients without hypertension. A study from Taiwan suggests that there is a strong positive association between IOP and SBP and DBP in males [31]. Moreover, two studies conducted in China showed that higher SBP is significantly associated with increasing IOP [13,39]. There are many studies that share the same results [10,36,40,41]. Similarly, IOP and diabetes were found to have a strong positive relationship in several studies [37,42-45]. The link between glucose and IOP may be explained by the autonomic dysfunction in diabetes and the osmotic gradient generated by a high blood glucose level, which results in a fluid shift into the intraocular space [35]. Elevated systemic arterial pressure raises the ciliary artery pressure and aqueous fluid filtration, both of which can lead to an increase in IOP. Furthermore, hypertension can cause a decrease in aqueous outflow and an increase in blood volume in the ciliary body [46].

This study's strength is that, to the best of our knowledge, this is the first study to evaluate the effect of systemic health parameters on IOP in the western region of Saudi Arabia. However, we faced some limitations such as our small sample size - a total of 255 records.

## Conclusions

Our study could not significantly prove the relation between IOP and the following: sex, BMI, LDL, HDL, TG, and total cholesterol levels. We suggested further evaluation in a larger population group with a larger sample size due to the immensity of this issue. For instance, although Saudi Arabia has a high prevalence of obesity, there are few studies evaluating the association between IOP and systemic health parameters in Saudi Arabia.
